# Lecture on the Local Effects of General Diseases

**Published:** 1860-11

**Authors:** Claude Bernard


### LECTURE ON THE LOCAL EFFECTS OF GENERAL DISEASES.

BY CLAUDE BERNARD.

That local affections, when unattended by general symptoms, cannot be viewed in the light of real diseases, and that the vessels or nerves are invariably the agents through which disorders, engendered upon a single point, are enabled to pervade the entire organism, has, we believe, been proved in our last lecture beyond the possibility of a doubt.

The various glands disseminated throughout the system have, as you are well aware, been divided into two great classes; those which extract from the blood certain special principles, which communicate to each particular secretion its own individual properties; and those which, on the contrary, appear to secrete the blood itself, if I may be allowed to use the expression, or, in other words, to enrich the circulating fluid with the products elaborated within their own tissue. Such, at least, are the views which have justified several physiologists in placing by the side of ordinary glands, the spleen, the thymus, the supra-renal capsule, and other highly vascular organs not provided with an excretory duct. The lungs, in which the great process of oxygenation is accomplished, exhibit, in its full force, the power attributed to this second order of glands; and the liver, which, if the biliary secretion is alone considered, belongs to the first class, is at the same time included in the second, on account of the grape-sugar which it produces. Now the influence of moter nerves on secretion is universally acknowledged; while the sensitive fibres are known to enjoy the property of setting in motion the opposite system; we are, therefore, naturally led to the conclusion that affections confined, at the outset, to one particular spot, may frequently poison the blood through a vitiated secretion of its fundamental elements, and thereby exert a deleterious influence over the whole system.

But diseases, however general in their nature, must always be viewed in connection with material alterations occurring within the body. The immortal writings of Bichat have taught us to look upon the vital powers not as forces independent of matter, but as the mere natural results of the properties enjoyed by matter, when impregnated with life; in this manner does the contractile force, in muscles, arise, as Haller expressed it, from the power of contraction possessed by each individual muscular fibre; and, in this manner, are the general properties of the nervous system to be referred to the faculties bestowed on each individual filament. The old notions on this subject are, however, recalled by the name of vital *powere*, which Bichat still employed for the purpose of expressing the properties of living organic matter; and in the use of words alone does he follow the example of the ancients, whose views on the subject were entirely different from his own. Since that moment the labors of our contemporaries have invariably followed the same direction Pinel overthrew the doctrine of essential fevers, and struggled to prove that anatomical lesions were invariably their cause, however difficult it may be to discover them in certain cases. Broussais upheld the same opinions, in his various chapters on hectic fever; and science is now pursuing the same track; in the properties of matter known do we seek the cause of physical phenomena, whether belonging to organic or inorganic bodies. It, however, we endeavor to advance a step further, and to examine the intimate nature of the properties with which matter is endowed, we are at once brought to a stop; to solve the problem is not within the power of human reason.

There exists, therefore, no disease whatever which is not connected with a local injury; but after firmly establishing this great principle, we are often at a loss to determine whether the disorders which occur for notice, in the organs of patients who die of certain maladies, are the cause or the results of the disease; thus, for instance, the hypertrophy of the spleen which usually accompanies intermittent fever, and the intestinal ulcerations which coincide with typhoid affections, are held by some physicians to be the cause, by others, the mere consequence of the disorder. Physiology shows us that in the majority of cases, morbid alterations are the effects of sickness instead of being the hidden springs which gave it birth. Let us recall to mind for a single moment, the symptoms of the disease artificially provoked by injecting sand into the capillary vessels; an intense fever shortly makes its appearance, through the mere effects of nervous sensibility; now, in this case, the fever is not *sine materia*; a well-known cause has called it into play: yet, under these circumstances, what modification has occurred within the nervous system? Who shall teach us the difference which exists between a nerve in a state of rest, and a nerve in a state of intense irritation, from outward agents? Let us not, however, be too much ashamed of our ignorance in this respect; it is no disgrace to the Medical Sciences; in fact, no branch whatever of human knownledge is totally exempt from similar difficulties: is there a natural philosopher who will undertake to assign the difference between the molecular arrangement of a magnet, and that of an ordinary bar of steel? The inference to be drawn from these reflections is, that we are never entitled, even in the most emparrassing cases, to admit the existence of a physiological symptom which does not result from some material cause; but the imperfections of our actual knowledge, on the one hand, and those of the human mind on the other, render it impossible to seize in every case the mutual relation which exists between them.

The consequences of disease are, therefore, various anatomical lesions, which must not be viewed as the starting-point of the morbid series. In eruptive fevers the only characteristic lesion with which we are acquainted is a cutaneous disorder; no one, however, would think of describing the variolic pustulæ as the hidden source of the predominant affection. We are thus led, by a natural train of reasoning, to inquire in what manner general disease becomes local, after having ascertained the process by which local diseases become general: the two questions are most intimately connected.

A poison being introduced into the economy, the animal dies; a series of morbid phenomena have been produced the the explanation of which is not always forthcoming when the autopsy is made. But in the easiest of all cases—that of fever—is not a simple wound capable of producing the disorder? And if the animal happens to be ill-disposed, does it not sometimes die from the general reaction provoked? and are not local alterations (pneumonia, for instance,) often found to exist? We have even ascertained that in order to make any given organ the seat of the disease, nothing more than the section of the corresponding branches of the sympathetic nerve was requisite; we therefore discover, in this very simple morbid series, two separate terms. In the first place, a local affection which becomes general; and, in the second, a general disease which fixes upon one particular point. How can entirely local lesions, such as congestion, inflammation, and the formation of pus, be most conveniently explained when arising from general causes? The circulation has evidently been put out of order; and it has been presumed that the sympathetic nerve, so intimately connected with vascular physiology, is the channel through which the morbid impulsion is transmitted. Now, when inflammation commences, the capillaries become the seat of peculiar phenomena; circulation is at first accelerated, and afterwards retarded; fibrin accumulates in in the little vessels and fills them up; and the point on which the obstruction arises, is known to be the primitive seat of inflammation. The buffy coat which, after blood has been drawn, rests on the surface of the clot, is a characteristic symptom in all inflammatory diseases. But how is its formation to be explained? what prevents the blood from coagulating as in a healthy state? A change has evidently taken place in the density of the fibrin which it contains, since, instead of imprisoning the globules, as usual, in its meshes, it rises to the surface of the liquid, in consequence of its lesser specific gravity. For this reason alone is the clot formed in an unusual manner; for it seems highly probable that the fibrin dissolved in the blood, the density of which it increases, is intended to hold in suspension the blood corpuscles; in fact, we see these little bodies fall to the bottom of a basin containing defibrinated blood, leaving the serum entirely colorless. It therefore may be advanced that in inflammations the fibrin contained in the blood has grown lighter than usual, since, when the fluid drawn from the veins is allowed to rest, it rapidly rises to the surface.

When the capillary circulation is examined under the microscope, the globules are seen occupying various positions; some of them adhering to the sides of the vessel, others rapidly moving in the sanguine torrent. After injecting into the arteries a large amount of defibrinated blood, M. Poiseuille saw the blood corpuscles form, as it were, a precipitate in the little vessels, as if obeying the ordinary laws of gravitation; and obstructions of greater or less extent, were, in this manner, rapidly formed. Supposing no special predisposition to exist in the economy, the liver and lungs would, of course, become the principal seat of similar impediments; the vascular organization of their tissue explains the preference. By this simple train of reasoning we can easily understand the creation of vascular deposits in various points of the body, under the influence of a modification in the physical properties of fibrin, when inflammation is produced; but when the blood ceases to be in motion in the centre of our tissues, the temperature of which is always sufficiently elevated to favor the action of chemical affinities, a putrid decomposition of the globules soon takes place; the assertion may easily be proved, by allowing defibrinated blood to stagnate for a short time in a basin, under a temperature of 95 degrees. The blood corpuscles are collected at the bottom of the recipient; the serum in the upper parts remains therefore unaltered; but let a small quantity of the same liquid be drawn, with a glass tube, from the immediate vicinity of the globules, and it will be found to contain both sulphuretted hydrogen, and other products of decomposition.

Let us now inquire what is likely to result from the accumulation of putrid deposits within the principal organs of the economy; a local irritation will, in the first instance, be produced, which, acting through the sensitive nerves upon the system as a whole, brings on general symptoms again; let us add to these the poverty of the blood, which an imperfect nutrition no longer supplies with its proper elements, and the presence of deleterious substances arising from putrefaction, within the torrent of circulation; and it will astonish no one to see so important an assemblage of morbid phenomena ultimately end in death. Thus we find that a complete series of morbid symptoms frequently arises from an entirely local irritation, and follows out its natural course when once the starting point is given.

But fever is not, after all, the only mode in which general affections are enabled to bring on local disorders. In eruptive diseases, for instance, another totally different cause may be supposed to act. Let us inject, for instance, a large dose of carbonate of ammonia into the animal's veins; after experiencing, for a time, considerable uneasiness, the animal recovers, the noxious substance having been gradually eliminated. In this case, we find an instance of a general disease without any local disorder; but we are aware, at the same time, that poisons, however widely distributed throughout the economy produce local effects; phosphorus affects the lungs, and cantharides the bladder; and the peculiar mode in which these alterations take place is well known to you, namely: the elimination of the toxic agent through special organs. Now, in eruptive fevers, an entirely similar process takes place; an irritating substance is expelled by the skin, and leaves there deep marks of its passage; here, then, we have an action of a purely mechanical character, which does not offer the slightest analogy with those we have just referred to.

It now remains for us, gentlemen, to inquire into the influence exerted by exterior agents upon the histological elements of living tissues, and the modifications to which they give birth; and, in pursuing these investigations, we shall be struck with the differences exhibited by the various organs, whether considered in the various degrees of the animal scale, or in animals of the same species, or even in one and the same individual, when one apparatus is compared with another; and after having ascertained to what extent, and with how great intensity they are acted upon by medicinal agents, we shall be prepared to examine the process by which medical influence is enabled to struggle against disease, and sometimes to overpower it.—*Med. Times and Gazette.*

---

Year Book of American Contributions to Medical Science and Literature.—Dr. O. C. Gibbs proposes to issue a yearly volume with the above title. Each volume is to be divided into three parts; Part First is to contain a summary of all the important and original papers found in the American journals for the preceding year. Part Second will contain a summary of all papers found in the published transactions of the National and various State and County Medical Societies. Part Third, will embrace reviews of all Medical Books of American authorship, published during the year, with a summary of all the novelties in opinion or practice therein. Those who wish to obtain the work—to be issued in January, 1861—should send their names and $3—the subscription price—to O. C. Gibbs, M. D., Frewsburg, Chautauque county, N. Y.

---

An Editor's Appeal.—Dr. Benson, of the *Louisville Medical News*, urges his subscribers to pay up, finishing with this appeal, which we want some of our subscribers to read and profit by:

"If you are our friends you should sustain us by paying what you owe. If you are our enemies you should 'heap coals of fire on our head' by paying what you owe. If you are entirely indifferent to us, manifest, if you please, that indifference by refusing longer to take our journal from the office, first, however, having complied with the law and equity by paying what you owe. If you have not the money send postage stamps, prairie chickens, old Bourbon, knit stockings, counterpanes, wolves' scalps, buffalo hides, sheep pelts, coon skins, whale leather, cast-off clothing—anything but your 'best wishes for our success,' unsustained by evidence."

